# Intramuscular Ketamine Pharmacokinetics in Humans: A Review

**DOI:** 10.1007/s13318-026-01012-2

**Published:** 2026-05-29

**Authors:** Julian Ananyev, Todd M. Myers

**Affiliations:** United States Army Medical Research Institute of Chemical Defense, 8350 Ricketts Point Rd., Aberdeen Proving Ground, MD 21010 USA

## Abstract

For over 50 years, ketamine has been approved for anesthesia and indicated for analgesia. In recent years, new clinical uses of ketamine have become increasingly common and continue to emerge. Intramuscular administration is a main route of administration and is particularly important in situations where resources are limited, such as in emergencies and low-income areas. The goal of this review is to summarize the literature on the pharmacokinetics of intramuscular ketamine in humans. Ten studies assessing intramuscular ketamine pharmacokinetics were identified. These studies looked at doses between 0.1 and 6.0 mg/kg in both healthy volunteers and clinical patients, and in adults and children. The pharmacokinetics of intramuscular ketamine were found to be dose-dependent, with maximal plasma concentrations linearly increasing from 27 ng/mL at a dose of 0.1 mg/kg to 1970 ng/mL at a dose of 6.0 mg/kg. The time to maximal plasma concentration ranged from 10 to 30 min and was largely uncorrelated with dose. The bioavailability of intramuscular ketamine remains unclear, with reported values of 41.1–93.0%. Children displayed faster absorption than adults, and possibly higher and faster norketamine concentrations, but otherwise similar ketamine kinetics. Additional pharmacokinetic data, especially at higher doses of ketamine (> 1.0 mg/kg), are needed to better inform dosing in emerging and diverse clinical uses and scenarios.

## Key Points

**Table:** 

Intramuscular ketamine maximal plasma concentrations are dose-dependent, whereas the time to reach maximal plasma concentration ranges from 10 to 30 min and is uncorrelated with dose.
Intramuscular ketamine bioavailability is unclear, with reports ranging from 41.1 to 93.0%.
Little pharmacokinetic data exists for intramuscular ketamine, especially for doses greater than 1.0 mg/kg which are clinically relevant for sedation and the management of status epilepticus.

## Introduction

Ketamine is a common anesthetic agent used in a variety of settings. It is a noncompetitive *N*-methyl-d-aspartate and glutamate receptor antagonist which also targets other diverse receptors, including the mu-opioid receptor. Ketamine is indicated as a sole anesthetic agent for procedures not requiring skeletal muscle relaxation, induction of anesthesia prior to the administration of other general anesthetic agents, and to supplement low-potency agents [1, 2]. The drug has an excellent safety and efficacy profile after acute use and has a very high therapeutic index [3–11]. In accidental ketamine overdoses in the hospital setting, doses 5–100 times the intended dose led to no adverse outcomes, usually just prolonged sedation [12–14].

Ketamine also has a number of off-label uses [2, 3, 15, 16]. Namely, it is often used in emergency situations for chemical sedation [10], and is highly effective for pain management [2]. It has additional emerging use for the treatment of depression and other psychiatric conditions [2, 6–8, 15, 17, 18], and shows early evidence for efficacy in immune modulation, tumor inhibition, preventing neuronal cell death after traumatic brain injury and strokes, and bronchodilation during asthma [15]. Recent attention has shifted to assessing ketamine as an anticonvulsant, especially in the treatment of benzodiazepine refractory status epilepticus [19, 20]. Outside the clinical use of ketamine, the drug is commonly taken recreationally for its dissociative effects [21].

A variety of routes of administration are available for ketamine, including intravenous, intramuscular, subcutaneous, intranasal, oral, sublingual, and rectal [22]. The intramuscular route is particularly relevant for emergency situations, when establishing intravenous access is difficult or time-consuming, and in low resource areas where intravenous fluid is expensive [23]. Intramuscular ketamine is a recommended treatment option by a joint commission including the American College of Emergency Physicians and National Association of EMTs, among other organizations, at doses of 50 mg for analgesia and 3…5 mg/kg for acute agitation/excited delirium [24]. Furthermore, the intramuscular administration route gains even more prominence in tactical medicine. The Tactical Combat Casualty Care guidelines recommend doses of 0.5–1.0 mg/kg for moderate to severe pain and 2–3 mg/kg for procedural sedation [25]. Since these guidelines have been implemented, military ketamine usage has increased significantly and has become the preferred combat analgesic [26–28]. Additionally, intramuscular ketamine has been used for psychiatric treatment and other various clinical presentations [29–33]. An especially exciting use is for terminating seizures and maintaining seizure-free neurological activity [19]. Intramuscular ketamine offers easy and quick administration in a variety of situations without the equipment, experience, and resources needed for intravenous administration.

The distribution and metabolism of ketamine are largely understood [34, 35]; however, limited evidence exists regarding the intramuscular route, particularly at higher doses [35]. The commonly cited intramuscular bioavailability of 93% [2], which helps inform dosing, is based on a study of just four volunteers (aged ~ 31.8 ± 2.0 years) and at the low end of the effective dose range (0.5 mg/kg) [36]. Understanding the intramuscular pharmacokinetics of ketamine in humans, especially the absorption, is vital to properly inform drug selection, route, dosing, patient monitoring, and establishing a baseline for drug interactions, in particular for emerging indications of ketamine.

The purpose of this narrative review is to summarize the pharmacokinetic properties of ketamine administered intramuscularly to inform practical clinical use, dosage, and illuminate knowledge gaps surrounding this important route of administration. Further, this work may assist in the development of new formulations and delivery methods of ketamine.

## Methods

A literature search was performed in August 2025, using the PubMed and EMBASE^®^ databases. Search terms included ‘ketamine’ and (‘pharmacokinetic’ OR ‘pharmacokinetics’ OR ‘PK’) and human and (‘intramuscular’ or ‘IM’). Date of publication was not filtered. Additionally, FDA product labels for ketamine products were hand searched for pharmacokinetic studies not reported in other published sources. Furthermore, Google Scholar (Google, Mountain View, CA, USA) was used to search for foreign-language studies not indexed to English databases. Google Translate (Google) was used to translate ‘Intramuscular ketamine pharmacokinetics’ to Spanish, Portuguese (Portugal), French, Italian, German, Turkish, Russian, Arabic, Hindi, Bengali, Chinese (simplified), Korean, and Japanese for these searches. Any foreign-language studies identified were translated to English via Google Translate for review. Finally, from all studies identified, the backward and forward snowballing techniques, using an article’s references or citations to the article, were used to identify additional works missed or not indexed.

This review included original, empirical research studies that reported prespecified pharmacokinetic parameters for ketamine given to human volunteers or patients via intramuscular injection at any dose. Studies using ketamine alone were preferred, but studies using the combination of ketamine with one or more other drugs were still open to inclusion. Both traditional and population pharmacokinetics studies were deemed appropriate for inclusion. Additionally, the pharmacokinetics of the metabolites of ketamine (norketamine, dehydronorketamine, hydroxyketamine, and hydroxynorketamine) following intramuscular administration of ketamine to humans were included in this review. The prespecified pharmacokinetic parameters include maximum plasma concentration (*C*_max_; a measure of peak drug exposure; ng/mL), time to maximum plasma concentration (*t*_max_; a measure of speed of onset; min), area under the plasma concentration-time curve (AUC; a measure of overall drug exposure; ng × min/mL), terminal elimination half-life (*t*_½_; a measure of drug cleared from blood; min), bioavailability (*F*; a measure of amount of drug reaching systemic circulation; %), total body clearance (CL; a measure of blood cleared of drug; L/h/70 kg), absorption rate constant (*K*_a_; rate at which drug enters blood; 1/min), volume of distribution (*V*_c_ for central and *V*_c_ for peripheral; a measure of drug found in body relative to blood liters; L/70 kg), and any reporting of plasma concentrations of ketamine or norketamine over time (ng/mL). From each study, measures of central tendency and variability were extracted, when available, and summarized. Plasma concentrations presented in figures were determined using the ImageJ open-source software by setting the *y*-axis as a scale, measuring the distance of the point from the *x*-axis, and calculating the plasma concentration based on this measurement [37, 38]. Pharmacokinetic parameters presented in units differing from those described above were converted, as appropriate. The pharmacokinetics of adults and children were separated due to potential physiological differences.

## Results

Ten studies, with a total of 113 participants receiving intramuscular ketamine, were included in this review [17, 18, 36, 39–45]. Table 1 provides a summary of study characteristics. Pharmacokinetic data were obtained from traditional pharmacokinetic studies using doses of 0.1 [18], 0.2 [18], 0.3 [18], 0.4 [18], 0.5 [36, 40, 41, 43, 45], 1.0 [41, 43, 45], and 6.0 mg/kg [39, 42] of ketamine. Population pharmacokinetic studies built their models on collected doses between 0.1–0.4 mg/kg [17] and 3.0–5.0 mg/kg [44], respectively. The population pharmacokinetic study using higher doses also extended their model to simulate intramuscular doses between 2.0 and 10.0 mg/kg. A majority of these studies used ketamine alone. Two studies using high doses of ketamine for sedation during surgery premedicated patients beforehand (children received 3 mg/kg of oral trimeprazine tartrate; adults received 2 mg of oral lorazepam) [39, 42]. One study gave ketamine for analgesia as needed following tonsillectomy surgery in which patients were premedicated with 10–15 mg of intramuscular diazepam and anesthetized with 2 mg of alcuronium chloride and 3–5 mg/kg of thiopental. Endotracheal intubation was performed under oxygen ventilation following administration of 2 mg/kg of succinylcholine. Anesthesia was maintained using 66% nitrous oxide, 33% oxygen, and halothane or enflurane [41]. These studies were held worldwide, in Scotland, Germany, Singapore, and Australia, with a majority using patients and only one study using healthy volunteers [36, 40]. Results for the reported pharmacokinetic parameters from each study are available in Table 2 and plasma concentration over time is presented in Table 3.

**Table 1:** Summary of included studies

Study (year)	Ketamine dose (s)	Pharmacokinetic design	Location	Sample size and characteristics	References
Nimmo and Clements (1981)	6 mg/kg (location unspecified)	Traditional	Scotland	3 adult patients undergoing surgery	[39]
Grant et al. (1981)	0.5 mg/kg (triceps muscle)	Traditional	Scotland	6 healthy adult volunteers	[36]
Clements et al. (1982)					[40]
Hirlinger et al. (1983)	0.5 mg/kg (deltoid muscle)	Traditional	Germany	3 patients following tonsillectomies	[41]
	1.0 mg/kg (deltoid muscle)			9 patients following tonsillectomies	
Grant et al. (1983)	6 mg/kg (thigh)	Traditional	Scotland	5 child patients undergoing minor elective surgery	[42]
Hirlinger and Dick (1984)	0.5 mg/kg (deltoid muscle)	Traditional	Germany	7 prehospital trauma patients	[43]
	1.0 mg/kg (deltoid muscle)			6 prehospital trauma patients	
Brandt and Dick (1989)	0.5 mg/kg (deltoid muscle)	Traditional	Germany	10 prehospital trauma patients	[45]
	1.0 mg/kg (deltoid muscle)			10 prehospital trauma patients	
Hornik et al. (2018)	3.0–5.0 mg/kg (location unspecified)	Population	Singapore	50 child patients for sedation in emergency department	[44]
Loo et al. (2016)	0.1–0.4 mg/kg (deltoid muscle)	Traditional	Australia	4 adult patients with major depressive disorder	[18]
Abuhelwa et al. (2022)		Population			[17]

**Table 2:** Summary of reported intramuscular ketamine pharmacokinetic parameters

Dose (mg/kg)	*n*	Age	Gender (% male)	Weight (kg)	C_max_ (ng/mL)	*t*_max_ (min)	AUC (ng × min/mL)	*t*_½_ (min)	*F* (%)	CL (L/h/70kg)	*K*_a_ (1/min)	*V*_c_ (L/70kg)	*V*_p_ (L/70kg)	References
0.1–0.4	4	45.6^a^	80^a^	81.8^a^	27–190^a^	–	–	–	64.4^c^	69.6^e^	0.109^c^	79.3^e^	87.4^e^	[17, 18]
0.5	6	31.8	–	70.7	240	22	23.6	155	93.0	97.44	0.167	–	–	[36, 40]
	3	Adult	–	–	147^a^	10^a^	–	–	–	–	–	–	–	[41]
	7	31^a^	100	65^a^	147	30^a^	–	–	–	–	–	–	–	[43]
	10	32.1	80	79.0^a^	65^a^	30^a^	–	–	–	–	–	–	–	[45]
1.0	9	Adult	–	–	254^a^	15^a^	–	–	–	–	–	–	–	[41]
	6	29^a^	70^a^	71^a^	474	15^a^	–	–	–	–	–	–	–	[43]
	10	33.8	90	62.4^a^	188^a^	30^a^	–	–	–	–	–	–	–	[45]
3.0–5.0	50	3.7^b^	38	14.9^b^	–	–	–	–	41.1	38.9^d^	0.042^d^	32.8^d^	152^d^	[44]
6.0	5	6.9	100	18.9	–	–	–	–	–	–	–	–	–	[42]
	3	40.66	33.3	63.66	1970	20	–	–	–	–	–	–	–	[39]

Presented as means unless otherwise indicated. – Data not available

*AUC* area under the curve, *CL* total body clearance, *C*_*max*_ maximum plasma concentration, *F* bioavailability (as compared to intravenous data), *IM* intramuscular, *IV* intravenous, *K*_*a*_ absorption rate constant, *N* sample size, *SC* subcutaneous, *t*_½_ terminal elimination half-life, *t*_*max*_ time to maximum plasma concentration, *V*_c_ central volume of distribution, *V*_*p*_ peripheral volume of distribution.

^a^Approximation based on best available source information

^b^Median

^c^IM data combined with SC data by the original study authors to calculate this parameter

^d^IM data combined with IV data by the original study authors to calculate this parameter

^e^IM data combined with SC and IV data by the original study authors to calculate this parameter

**Table 3:** Plasma ketamine and norketamine concentrations (in ng/mL) with time following intramuscular ketamine administration

Substance	Age	Dose (mg/kg)	Time after ketamine injection (min)																References
			3	5	10	15	20	30	45	60	90	120	180	240	300	360	420	540	
Ketamine	Adult	0.5	–	~ 159	~ 176	~ 195	~ 189	~ 158	~ 122	~ 96	–	~ 55	–	~ 45	–	–	~ 10	–	[36]
			~ 12	~ 100	~ 150	~ 150	–	~ 124	~ 109	~ 98	~ 102	–	–	–	–	–	–	–	[41]
			–	~ 108	~ 120	~ 140	–	~ 149	–	–	–	–	–	–	–	–	–	–	[43]
			–	~ 39	~ 46	~ 52	–	~ 65	–	–	–	–	–	–	–	–	–	–	[45]
		1.0	~ 44	~ 166	~ 233	~ 258	–	~ 245	~ 196	~ 134	~ 107	~ 96	–	~ 50	–	~ 39	–	–	[41]
			–	~ 360	~ 440	~ 473	–	~ 372	–	–	–	–	–	–	–	–	–	–	[43]
			–	~ 152	~ 173	~ 161	–	~ 188	–	–	–	–	–	–	–	–	–	–	[45]
		6.0	–	440	–	1710	1970	1730	–	1440	–	–	700	–	–	–	–	> 150	[39, 42]
	Child	6.0	–	2090	–	2150	–	1950	–	1270	–	–	500	–	–	–	–	–	[42]
Norketamine	Adult	0.5	–	~ 5	~ 6	~ 19	~ 35	~ 56	~ 75	~ 79	–	~ 78	–	~ 58	–	–	~ 29	–	[36]
		1.0	–	~ 16	~ 32	~ 76	–	~ 128	~ 159	~ 146	~ 138	~ 142	–	~ 73	–	~ 68	–	–	[41]
		6.0	–	0	–	83	–	193	–	422	–	–	472	–	–	–	–	–	[42]
	Child	6.0	–	37	–	279	–	546	–	695	–	–	658	–	–	–	–	–	[42]

– Data not available

~ Approximation using ImageJ (image measuring tool)

### Adults

In the 1980 s, Grant et al. and Clements et al. studied the bioavailability, pharmacokinetics, and analgesic activity of ketamine in healthy volunteers, considering three routes of administration: intravenous (IV), intramuscular (IM), and oral (PO) [36, 40]. The two referenced articles present pharmacokinetic data from the same patients during the same experiments, just in slightly different ways. The group found the following results using a sample of six individuals (31.8 ± 2.0 years old; 70.7 ± 4.4 kg), administering IM ketamine in the triceps muscle at a dose of 0.5 mg/kg and using a two compartment model: *C*_max_ (240 ± 50 ng/mL [36] or 243 ± 49 ng/mL [40]), *t*_max_ (22 ± 4 min [36, 40]), bioavailability (93.0 ± 2.6% [36, 40]), and AUC (23.6 ± 2.2 ng × min/mL [40]). For a complete summary of pharmacokinetic parameters, please refer to Table 2. Notably, the bioavailability determination was from only four individuals by using area under the curve differences between IM and IV ketamine in the same patients. These papers also studied the pharmacokinetics of norketamine, finding the following: *C*_max_ (90 ± 10 ng/mL [36] or 92 ± 10 ng/mL [40]), *t*_max_ (77 ± 14 min [36, 40] or 78 ± 14 min [36, 40]), and AUC (31.3 ± 3.8 min × ng/mL [40]).

A brief study from the same group, published only as an abstract in the *Proceedings of the Anaesthetic Research Society* London Meeting of 1980 [39] and later referenced with additional data in a study comparing IV and IM ketamine pharmacokinetics in children and adults [42], looked at the pharmacokinetics of IM ketamine in three adults (one male; ages 24, 29, and 69 years; weights 74, 55, and 62 kg) at a dose of 6 mg/kg. They found the following: *C*_max_ (1970 ng/mL [39]) and *t*_max_ (20 min [39]).

To our knowledge, previous literature looking to characterize ketamine pharmacokinetics [34, 35] missed several key studies performed in the 1980 s, likely due to the fact these studies are published in German and the only English translations available are the abstracts indexed to PubMed [41, 43, 45]. Between the three studies, this group ended up determining the pharmacokinetics of IM ketamine in the deltoid muscle for 20 patients at a dose of 0.5 mg/kg (3 patients in their first study [41]; 7 in their second study [43]; 10 in the final study [45]) and 25 patients at a dose of 1.0 mg/kg (9 in first study [41]; 6 in second study [43]; 10 in the final study [45]). Of note, these studies looked at the pharmacokinetics in real patients. The first study gave ketamine for pain management following tonsillectomy (collecting blood prior to administration, and at 15, 30, 45, 60, and 90 min post-injection; two participants had blood drawn for up to 6 h, which also included norketamine and hydroxynorketamine quantification), whereas the second and third studies gave ketamine for pain management in the prehospital setting (by paramedics) for trauma, such as second-degree burns, peripheral fractures, and painful bruises or soft tissue injuries (collecting blood prior to administration, and at 5, 10, 15, and 30 min post-injection). These studies do not present direct pharmacokinetic variables (of the prespecified pharmacokinetic variables, they only present plasma concentrations over time), but the overall results echoed the findings from the Grant and Clements groups. Peak plasma concentrations occurred between 10 and 30 min after injection in all groups and were 100–300 ng/mL higher in the 1.0 mg/kg groups than in the 0.5 mg/kg groups. If plasma concentrations were dose-dependent, then doubling the dose should double the *C*_max_, and in these studies the *C*_max_ increased by a ratio of 1.72, 3.17, and 2.89 (average = 2.59).

Several recent studies by Loo et al. and Abuhelwa et al. looked at the pharmacokinetics of IM, subcutaneous (SC), and IV ketamine, specifically for its potential use in the treatment of depression [17, 18]. The first study only reported the *C*_max_ across different doses (0.1–0.4 mg/kg, administered in the deltoid muscle to four participants) and found a linear relationship between dose and *C*_max_ (~ 27 ng/mL at 0.1 mg/kg and ~ 190 ng/mL at 0.4 mg/kg) [18]. Their second study used the ketamine pharmacokinetics data from their first study to build a population pharmacokinetics model. They combined data from IM, SC, and IV administration for their modeling and got the following pharmacokinetic parameter estimates: CL (69.6 L/h/70kg), *K*_a_ (0.109 1/min), *V*_c_ (79.3 L/70kg), and *V*_p_ (87.4 L/70kg) [17]. Of note, the bioavailability of ketamine from the combined IM/SC routes was determined to be 64.4% [17].

### Children

Two studies considered the pharmacokinetics of IM ketamine in children. Grant et al. compared the pharmacokinetics of IM ketamine at a dose of 6 mg/kg between adults (see above) and children (*n* = 5; all male; mean age of 6.9 years; mean weight of 18.9 kg) [42]. They found that absorption of ketamine appeared to be faster in children than adults, but that there were no major differences in plasma ketamine concentration following absorption (see Table 3).

Hornik et al. sought to determine the population pharmacokinetics of IM and IV ketamine in children [44]. A sample of 50 children (98% Asian; 38% male; mean age of 3.7 years; mean weight of 14.9  kg) all received one IM dose between 3 and 5 mg/kg (median of 4.0 mg/kg). All but one subject contributed three pharmacokinetic samples (10, 30–60, and 120 min), and the authors used a two-compartment model with first-order absorption and elimination imputed from the IM and IV data. IM ketamine plasma concentrations were simulated for doses between 1 mg/kg and 10 mg/kg (1, 2, 4, 6, 8, and 10 mg/kg) for children of different weights (6–56 kg). Doses of 1 mg/kg reached *C*_max_ ~ 100–130 ng/mL with a *t*_max_ of ~ 20–30 min. Doses of 10 mg/kg reached *C*_max_ ~ 1130–1500 ng/mL with a *t*_max_ of ~ 20–30 min. They built a pharmacokinetic model with data from IM and IV administration and obtained the following parameters: CL (38.9 L/h/70 kg), *K*_a_ (0.042 1/min), *V*_c_ (32.8 L/70 kg), and *V*_p_ (152 L/70 kg). The bioavailability of IM ketamine was determined to be 41.1% using a population approach [44].

**Fig. 1:**
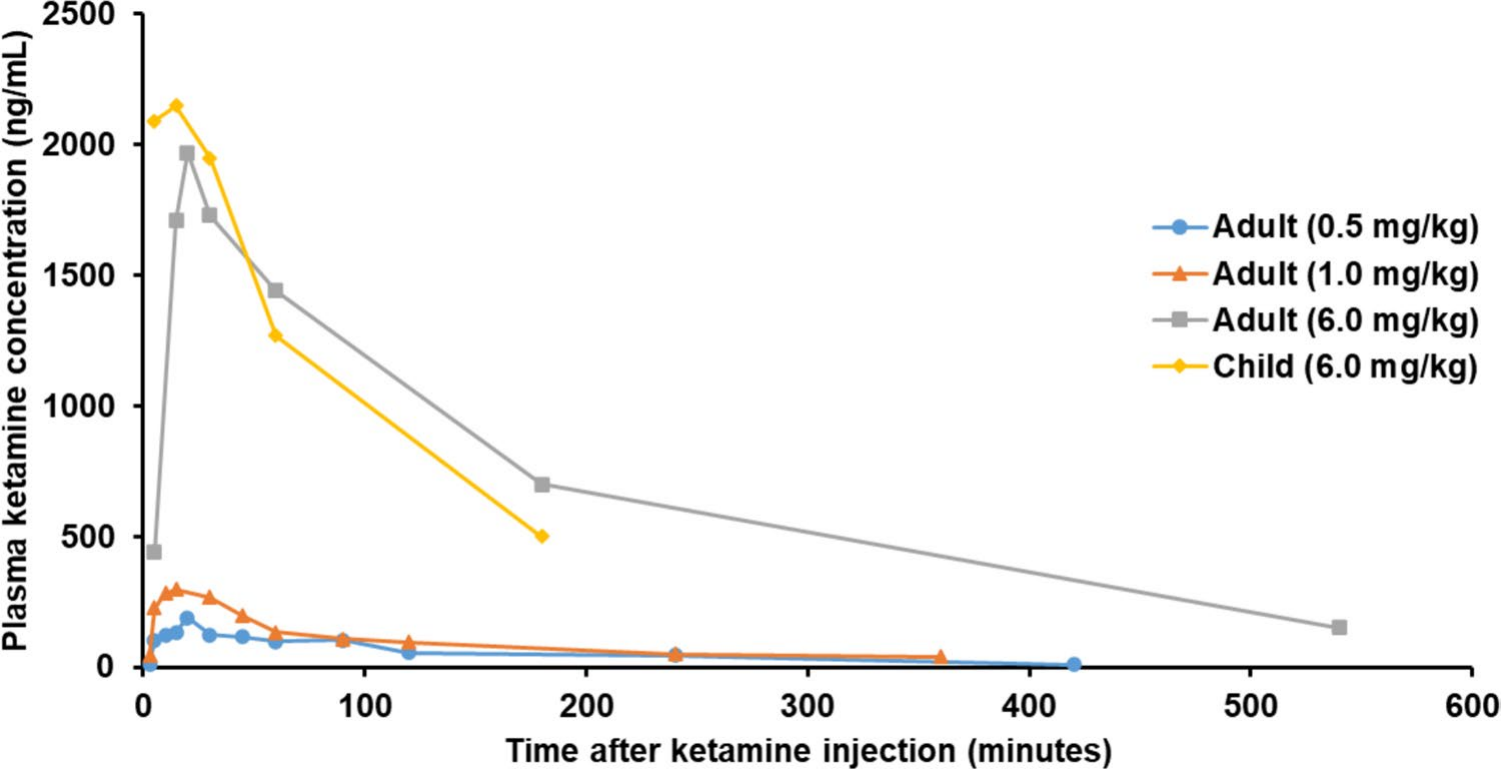
Average plasma ketamine concentrations across time. Reported plasma concentrations at each time point were averaged between studies at each dose/age group to produce concentration-time curves. Doses of 0.5 mg/kg in adults elicited maximal plasma concentrations (*C*_max_) of ~ 189 ng/mL and time to maximal plasma concentrations (*t*_max_) of ~ 20 min (*n* = 4 studies; [36, 41, 43, 45]). Doses of 1.0 mg/kg in adults elicited *C*_max_ of ~ 297 ng/mL and *t*_max_ of ~ 15 min (*n* = 3 studies; [41, 43, 45]). Doses of 6.0 mg/kg in adults elicited *C*_max_ of 1970 ng/mL and *t*_max_ of 20 min (*n* = 1 study; [42]), while doses of 6.0 mg/kg in children elicited *C*_max_ of 2150 ng/mL and *t*_max_ of 15 min (*n* = 1 study; [42])

**Fig. 2:**
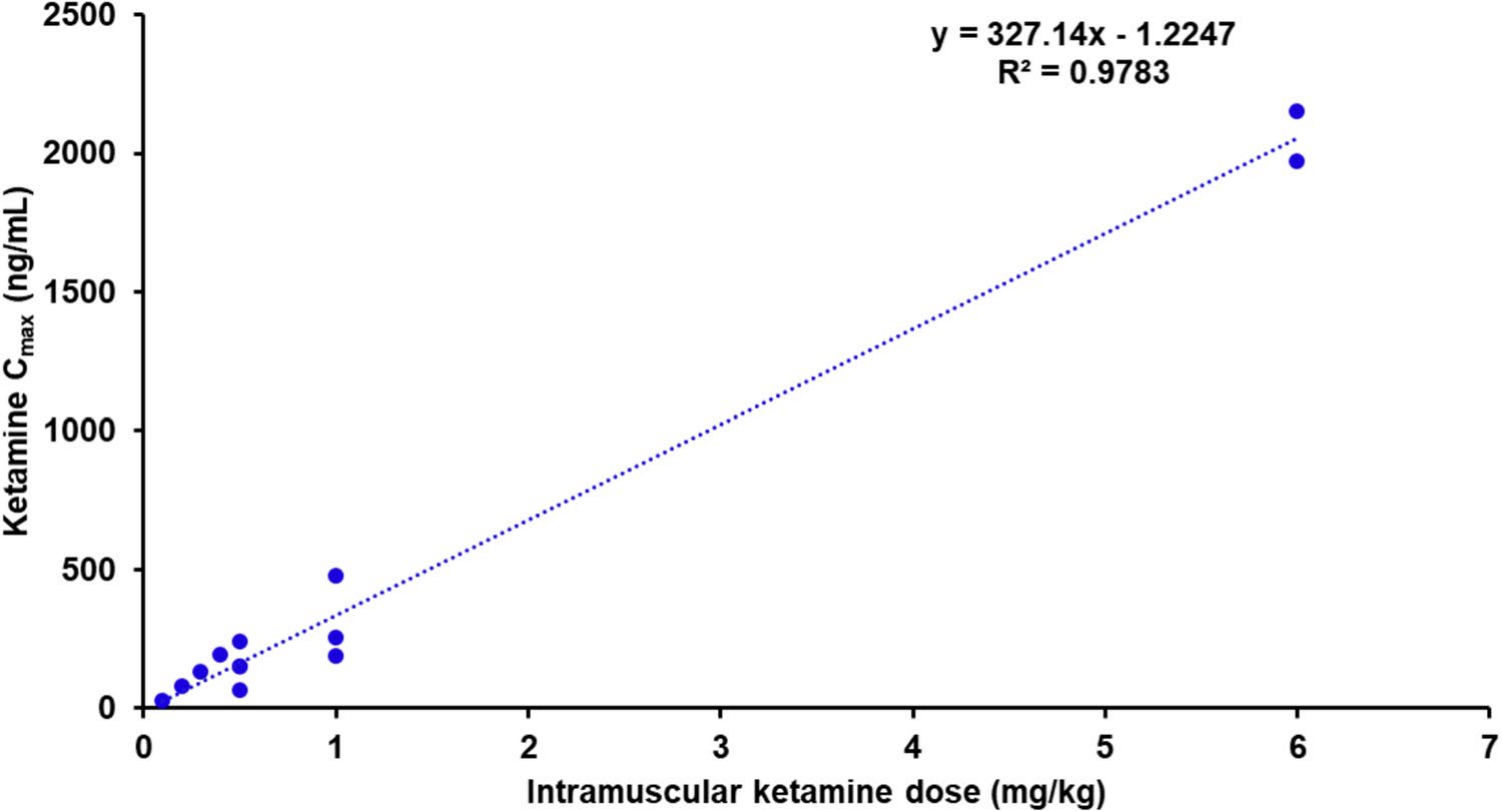
Maximal plasma ketamine concentrations (*C*_max_) as a function of intramuscular ketamine dose in different studies

**Fig. 3:**
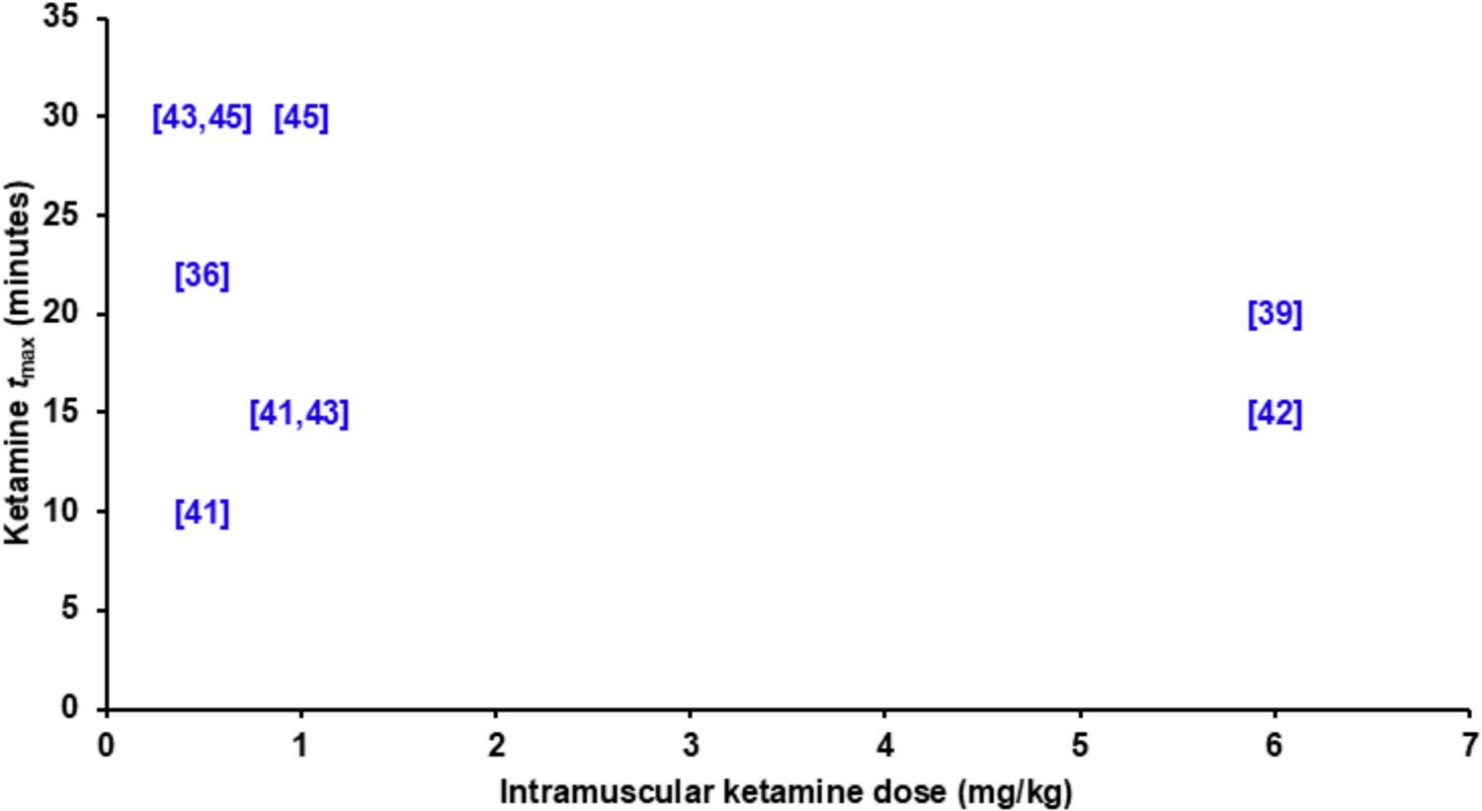
The time to maximal plasma concentration (*t*_max_) of ketamine following intramuscular administration at varying doses, as reported in different studies. *Reference numbers* indicate the corresponding studies, and the *data points* represent the mean values reported

In summary, ketamine pharmacokinetic profiles following intramuscular administration differed by dose. Figure 1 shows average ketamine plasma concentrations (across studies) over time at all reported doses. Multiple studies provided plasma concentrations for low doses of ketamine (0.5 and 0.1 mg/kg), whereas few studies reported data for higher doses. Ketamine is rapidly absorbed and eliminated fairly quickly. Important *C*_max_ data from across the available studies are summarized in Fig. 2. At these doses, the maximal plasma concentration of ketamine was dose-dependent. However, the time to reach maximal plasma concentrations ranged between 10 and 30 min and appears uncorrelated with dose. Figure 3 provides an overview of *t*_max_ outcomes from the included studies. Note that all studies reporting *t*_max_ of 30 min were performed in a prehospital setting and likely had a larger amount of methodological and patient variability.

## Discussion

Although ketamine was approved by the FDA for intramuscular injection over 50 years ago [15], few studies have examined its pharmacokinetics via this route. To our knowledge, a total of ten studies reported any pharmacokinetic parameters following intramuscular ketamine, and, of these, only three used doses relevant for sedation or anesthesia (> 1.0 mg/kg). Many of these studies contained a considerable amount of homogeneity in terms of participant demographics or did not report this information, compromising generalizability. Furthermore, only two of these studies used healthy volunteers, as opposed to clinical patients. Despite the paucity of quality pharmacokinetic data, meaningful conclusions can be drawn regarding maximal plasma concentrations and time to maximal plasma concentrations of ketamine.

The *C*_max_ of intramuscular ketamine ranged between 27 ng/mL at a dose of 0.1 mg/kg to 1970 ng/mL at a dose of 6.0 mg/kg in adults, with *t*_max_ ranging between 10 and 30 min. Notably, three studies reported bioavailability of the intramuscular route, and they all found very different values (93.0%, 41.1%, and 64.4%) [17, 36, 44]. Grant et al. compared area under the plasma concentration–time curves between four patients who received intravenous and intramuscular injections of 0.5 mg/kg of ketamine on separate occasions to determine a bioavailability of 93% [36]. This value has endured in the literature and continues to inform dosing recommendations, despite newer conflicting evidence [2]. Hornik et al. and Abuhelwa et al. both derived bioavailability from population pharmacokinetic models using variable dosing from 3.0–5.0 mg/kg and 0.1–0.5 mg/kg, respectively [17, 44]. The former study was in children and resulted in a bioavailability of 41.1%, while the latter study was in adults but was calculated from both subcutaneous and intramuscular data (although the authors claim that combining the bioavailability data between the two routes did not worsen model fit over separate bioavailability estimates) and resulted in a bioavailability of 64.4%.

The wide range of reported bioavailability values (41.1–93.0%) highlights the challenges in interpreting these results and underscores the need for a critical appraisal of the underlying methodologies. The studies differ substantially in their sampling schemes, pharmacokinetic analysis methods, and populations, which considerably limit the comparability of the reported estimates. For example, Grant et al. assessed the concentration–time profile over a 7-h observation period using a non-compartmental analysis, whereas Abuhelwa et al. used a 4-h observation interval but applied a more robust population pharmacokinetic model using nonlinear mixed-effects methods [17, 36]. Similarly, Hornik et al. employed a population pharmacokinetic model but with a relatively short observation interval of 2 h, which may have limited the accuracy of the bioavailability estimate [44]. Further, the collection of intravenous and intramuscular data in Hornik et al. occurred in separate clinical trials with variations in protocols. These methodological differences, combined with variations in dosing regimens, study populations (adults vs. children), and the inclusion of data from other administration routes, contribute to the observed discrepancies in bioavailability. Given these limitations, caution is warranted when interpreting and comparing bioavailability estimates across studies.

Intramuscular injection with higher doses of ketamine seems to lead to higher maximal plasma concentrations of ketamine and its primary metabolite norketamine. Between the studied doses of 0.1–6.0 mg/kg, *C*_max_ of ketamine increases dose dependently, whereas *t*_max_ ranges between ~ 10 and 30 min and appears uncorrelated with dose. Of course, norketamine peaks after ketamine and, based on the limited evidence, norketamine reaches maximal concentrations faster at lower doses of ketamine. In rats, norketamine has one-third the anesthetic and analgesic potency of ketamine, which may result in longer-lasting clinical effects following ketamine administration [46, 47]. Further, differences exist in pharmacokinetics between adults and children. There have been two studies in children, of which only one was comparative to adults. Children seem to have faster ketamine absorption and higher norketamine plasma concentrations, but an otherwise similar pharmacokinetic profile. Generally, children display faster intramuscular absorption than adults due to their lower muscle mass and higher perfusion at common sites of injection. Further, the metabolic activity of cytochrome P450 3A4 (CYP3A4) is greater in infants and children (especially during the first 6 months of life) than in adults, leading to potentially faster metabolism. Finally, children exhibit a faster plasma clearance and a shorter ketamine half-life than adults. However, it is important to note that during the first 3 months of life, the plasma clearance is decreased due to decreased metabolic capacity of the liver [34, 48].

The different routes of ketamine administration all have differing properties and considerations. Generally, ketamine has ~ 10–30% binding to plasma proteins and a high distribution. Ketamine is mainly metabolized into the active metabolite, norketamine, which is hydroxylated into 6-hydroxy-norketamine and then excreted in bile and urine after glucuronoconjugation. Ketamine elimination clearance is high (~ 12–20 mL/min/kg) [34]. The absorption of intravenous ketamine is rapid, with maximal plasma concentrations occurring close to 0 min after administration [35], and with quicker elimination than after intramuscular injection [19]. Intramuscular and intranasal administration both take around 20 min to reach maximal plasma concentrations [49]. Oral administration has the slowest absorption and also must undergo first-pass hepatic metabolism [35]. Intranasal and oral ketamine have bioavailability of 45–50% and 16–29%, respectively [35], which are both likely lower than the intramuscular route.

In particular, intranasal ketamine has gained significant attention in recent years as a practical alternative to other routes of administration for certain clinical scenarios. The intranasal route is well-suited for rapid, non-invasive analgesia or for treating psychiatric conditions such as depression and post-traumatic stress disorder, where ease of administration and quick absorption without the need for needles are clear advantages and increase patient satisfaction [50–52]. This route is especially useful in pediatric populations, where minimizing discomfort and distress is a priority [51]. In comparison, intramuscular ketamine is preferred for emergency situations and situations requiring high-dose, rapid-onset sedation, such as acute pain management in the emergency department, managing combative or agitated patients, or in the treatment of status epilepticus [53–55]. The intramuscular route achieves a faster onset of action, higher systemic drug exposure, more consistent dosing, and more pronounced pharmacodynamic effects than the intranasal route [51, 56]. While both routes have advantages, their selection depends on the clinical context, patient population, and desired therapeutic outcomes.

Clinicians must understand the intramuscular ketamine pharmacokinetic profile to better predict the time course of pharmacodynamic effects and safely optimize dosing. In humans, dissociative anesthesia is achieved with minimum peak plasma concentrations of 1200–2400 ng/mL [35], which may be achieved with a dose of 6 mg/kg, and possibly as low as 4 mg/kg. Awakening from anesthesia typically begins at plasma concentrations of 640–1100 ng/mL [35]. At a dose of 6 mg/kg, this can typically be expected around 100–200 min following intramuscular ketamine administration. Analgesia is associated with plasma concentrations of 70–160 ng/mL [35], which is typically achieved within 5 min at doses of 0.5 and 1.0 mg/kg and is achieved even quicker at a dose of 6.0 mg/kg. Analgesic effects can be expected to last for ~ 45 min at a dose 0.5 mg/kg, ~ 90 min at 1.0 mg/kg, and for more than 6 h at 6.0 mg/kg. Antidepressant effects require similar minimum peak plasma concentrations of 75–185 ng/mL [35]. Finally, the side effects of dissociation and psychotomimetic properties occur at around 100–250 ng/mL [35].

Intramuscular administration of ketamine is an effective option in many different clinical presentations. This route shows particular promise for emerging treatment options, especially in cases where intravenous access is difficult or longer elimination time is desired. One such indication is in the management of status epilepticus, where intravenous access is time-consuming and challenging, and a longer duration of ketamine elimination may prolong the duration of effective plasma concentrations necessary for seizure control. Further, military use of ketamine is rising [26–28], and is reducing reliance upon opioids in the prehospital setting [57]. The increasing medical utility of intramuscular ketamine may lead to advances in formulations and delivery methods. Of interest may be a ketamine autoinjector for simple and rapid injection, and devices or formulations (patches, microneedle arrays, or subcutaneous pumps, or encapsulated slow-release ketamine) offering a longer duration of effect (such as may be needed for prolonged field care, mass casualty, or battlefield scenarios, to control pain and/or status epilepticus).

### Limitations

Despite a comprehensive search of multiple databases, relatively few relevant studies were identified. In fact, seven of the ten identified studies are close to 40 years old. While this may raise concerns about the relevance of the data today, the pharmacokinetic principles applied in these studies are unlikely to have changed significantly over time, and the resulting parameters should be intrinsic to the drug’s properties. However, we acknowledge that advancements in analytical techniques, study designs, and clinical applications may have occurred since these studies were conducted, potentially altering the mathematical parameter estimates and outcomes. Further, there is a paucity of information across doses, with little pharmacokinetic data at higher dose ranges (> 1.0 mg/kg), yet higher doses are routinely used for sedation and anesthesia.

Additionally, methodology varied greatly across studies, such as differences in pharmacokinetic modeling, number and types of pharmacokinetic parameters collected, and site of intramuscular injection. Substantial variability in the reported bioavailability of intramuscular ketamine across studies, ranging from 41.1% to 93.0%, likely reflects differences in sampling durations, pharmacokinetic analysis methods, and study populations, which limit the comparability and generalizability of these findings. The current evidence is derived from diverse global locations (Europe, Singapore, and Australia) from healthy volunteers, prehospital, and hospital patients, but much remains to be investigated regarding potential differences in ketamine kinetics as a function of age, weight, sex, and in different clinical populations. Finally, language barriers in accessing and interpreting articles may have limited the amount and quality of pharmacokinetic data assessed.

## Conclusion

Ketamine is gaining prominence as a treatment option across diverse clinical scenarios, such as procedural sedation, analgesia, seizure management, and depression. The intramuscular route of administration offers distinct advantages in terms of ease of administration in variable situations, particularly in emergencies where time and resources are limited. The kinetics of this route are important to understand for proper dose selection. Peak plasma concentrations seem dose-dependent, whereas the time to peak plasma concentration remains constant between 10 min and 30 min. The intramuscular bioavailability is unclear. It is important to note that a scarcity of kinetic data exists using this route of administration. Only one study sample uses healthy volunteers, and it was completed at a very low dose of ketamine. More pharmacokinetic data, especially at doses greater than 1.0 mg/kg and in a more diverse sample of participants, is needed to better inform dosing and clinical application. Such improved data would aid not only in optimized use of current ketamine products but also in the development of improved devices and formulations.

## Declarations

### Availability of data and material

Data will be made available to interested parties upon request, as capable.
